# Determinants of dental caries in deciduous teeth among Iranian school children: Evidence from a developing country

**DOI:** 10.1371/journal.pone.0327141

**Published:** 2025-08-08

**Authors:** Bahareh Sadat Pezeshki, Awat Feizi, Bahareh Tahani

**Affiliations:** 1 Oral Public Health Department, Dental School, Isfahan University of Medical Sciences, Isfahan, Iran; 2 Department of Epidemiology and Biostatics, School of Public Health, Isfahan University of Medical Sciences, Isfahan, Iran; 3 Oral Public Health Department, Dental Research Center, Dental research Institute, Dental School, Isfahan University of Medical Sciences, Isfahan, Iran; Hamadan University of Medical Sciences School of Dentistry, ISLAMIC REPUBLIC OF IRAN

## Abstract

**Background:**

Understanding the social determinants of oral health is essential for addressing oral health disparities and facilitating the implementation of effective oral health policies. Therefore, the aim of this study was to assess the primary dentition caries status among 7-year-old children and to explore individual- and family-level associated factors.

**Methods:**

As part of a longitudinal study, this cross-sectional study was conducted between April 10 and May 8, 2024, in elementary schools in Isfahan, Iran. A valid and reliable self-administered questionnaire was distributed among 551 parents, including questions on demographic, socioeconomic, and psychological characteristics; oral health and dietary behaviors of their child; and some questions about the family status. Clinical examinations of the children were performed on-site at the schools. Based on the ICDAS II classification, six indices—including d₁–₂, d₃–₆, filled surfaces, missing surfaces, d₃–₆mfs, and d₁–₆mfs—were calculated. The correlation between factors was analyzed using Pearson and Spearman correlation coefficients. The multivariable associations of determinants were assessed using linear and logistic regression models (α = 0.05).

**Results:**

Finally, 417 children were examined. More than 90% of the children had dental lesions. Based on the multiple linear regression analyses, the mean d₁–₆mfs score was significantly higher in boys (B = 6.9; 95% CI: 2.8–11.2, P = 0.01) and children attending public schools (B = 11.35; 95% CI: 6.3–16.4, P < 0.01), and lower in children with a housewife caregiver (B = –6.33; 95% CI: –11.7 to –0.95, P = 0.021) and those with a healthier diet (B = –0.25; 95% CI: –0.48 to –0.02, P = 0.029). The mean d₃–₆mfs score was also significantly lower in children with a healthier diet (B = –0.29; 95% CI: –0.5 to –0.08, P = 0.006). Based on logistic regression analysis, the odds of having a filled tooth were significantly greater in girls (OR = 2.33; 95% CI: 1.51–3.59, P < 0.001), children attending private schools (OR = 1.91; 95% CI: 1.17–3.11, P = 0.01), and children whose caregivers possessed a high school diploma (OR = 1.9; 95% CI: 1.07–3.5, P = 0.048) or a university degree (OR = 2.47; 95% CI: 1.28–4.74, P = 0.007).

**Conclusion:**

The prevalence of tooth decay was high among children and was associated with demographic characteristics and socioeconomic status of the family, biological and behavioral traits of the child, and the knowledge and mental and emotional condition of the caregiver.

## Background

Globally, the burden of oral diseases is still high and they are the fourth most expensive diseases to treat. About 60–90% of schoolchildren are affected by dental caries, which causes millions of school days to be missed each year [1,2]. In 1990, approximately 22% of Iranian children’s deciduous teeth had caries [dmft = 4.37; 95% CI: 2.23–6.62]. Of these, over 83% were decayed, while less than 7% were filled [3]. Based on the last published national survey in 2017, the number of decayed, missing, and filled teeth (dmft) reached 5.03 (95% CI: 2.82–7.29). Notably, decayed teeth (dt) exhibited the largest increase, exceeding 17% over this period [3]. Moreover, it was reported that about 90% of 6-year-old children had experienced dental caries. Based on the Global Burden of Disease 2019, the estimated average prevalence of caries in deciduous teeth is 43%, and 69% of WHO Member States have a prevalence of more than 40%. In addition, more than three-quarters of cases of untreated caries in deciduous teeth are found in middle-income countries, due to inadequate resources to address the burden [4]. Pain, abscess formation, and the loss of teeth and their space in the jaw arch are typical consequences of tooth decay, resulting in reduced productivity and nutritional problems that may ultimately affect overall health and impair the quality of life [5].

Although dental caries is a multifactorial disease, it is largely preventable [6]. A confluence of sociopolitical, structural, and socioeconomic determinants—including social class, occupational position, education level, and income—can impact health outcomes. According to the World Health Organization’s Commission on Social Determinants of Health (CSDH), intermediary determinants such as neighborhood conditions, work environment, housing conditions, psychological state, and behavioral and biological factors further contribute to these health conditions [7].

Socioeconomic status has been proposed as an important predictor of caries in children [8–10]. It has been proven that a low level of parental education is associated with an increased prevalence of caries [8]. Other socioeconomic factors associated with oral diseases are low individual/household income [11]. In addition, the factors that cause dental caries among children are related to both the child and the family. Therefore, in order to foster optimal oral health in children, it is crucial to emphasize parental involvement in oral health education and dental care [12].

Studies have also established a relationship between more frequent brushing, especially using fluoride-containing toothpaste, and fewer new caries [9,10]. In addition, greater access to unhealthy consumer goods, such as foods and drinks with high sugar [13], and excessive carbohydrate intake significantly contribute to an increased incidence of dental caries [5,14].

Although many advances have been made in recent years to develop effective strategies that can improve oral and dental health [15], a comprehensive understanding of the social determinants of oral health is essential to address oral health disparities and facilitate the implementation of oral health policies and programs at local, national, and international levels [16]. Indeed, future policies should not simply address oral and dental health behaviors; rather, they should also focus on the political and economic drivers that create social inequalities (the structural determinants of oral health inequalities) [17]. Action on structural determinants is primarily the responsibility of national policy makers. However, the development of local and regional policies can be directed at the intermediary determinants of oral health inequalities particularly in developing countries such as Iran [7]. Targeted interventions that aim to develop and build individuals capacity are good examples; Oral health literacy programs that enhance core skills and competencies can facilitate and strengthen individuals’ ability to cope with adversity. Also, interventions that focus on developing supportive oral health environments in local settings such as schools and those that strengthen social networks will have beneficial effects on oral health outcomes [16]. Even the interventions aimed to change health behaviors should be directed at changing modifiable aspects of the environment to enable healthy choices to be easier at all stages of the life course [7].

In previous studies in Iran [18–20], the monitoring of the dental caries has been mostly relied on the DMFT (Decayed, Missing, and Filled Teeth) index that have some limitations in detecting early dental caries lesions and stage of progression. Furthermore, to the best of our knowledge, they mostly have been focused on some common determinants and none have applied the WHO Social Determinants of Health (SDH) framework. Given the importance of indicating the dental caries status using sensitive assessment indices and its determinants and also insufficient related research in Iran, this study aims to assess the caries status of children and its associated factors using the WHO SDH framework.

## Methods and materials

The study protocol was approved by the Ethics Committee in the Vice Chancellery of Research at the Isfahan University of Medical Sciences (IR.MUI.DHMT.REC.1403.021). In addition, informed written consent from the parents and then verbal consent from the children were obtained.

This cross-sectional study was conducted between April 10 and May 8, 2024, in public and private elementary schools in Isfahan, Iran. This research was part of a longitudinal study entitled: “Assessing cavity progression and the mean number of newly developed cavities in 7-year-old children, and their relationships with family socioeconomic status, child behavioral factors, and parental psychological factors over a one-year follow-up.”

Based on the baseline database from the main study, this analysis examined the prevalence and determinants of dental caries at various levels in the deciduous teeth of 7-year-old children in Isfahan

### Sample size and sampling method

The calculated sample size was 396, based on a significance level of 5% (Z = 1.96), a statistical power of 80% (Z = 0.84), and an assumed minimum odds ratio of 2.0 [12] for the relationship between the prevalence of dental caries and family socioeconomic status, child behavioral factors, and parental psychological factors.

This study employed a multi-stage cluster random sampling method. In coordination with the General Directorate of Education of Isfahan Province, three districts were randomly selected from six educational districts. Within each district, one or two public schools and one or two private schools were randomly selected, proportional to the population size of each district and the ratio of public to private schools. In each school, one or two first-grade classes of seven-year-old students were selected, ensuring gender balance between boys and girls. In order to observe ethical principles, all parents were asked to provide written informed consent prior to recruitment. Verbal assent was also obtained from the children. A package containing a parental questionnaire, a study description, and consent forms was distributed to parents through school administrators. One week later, in coordination with school officials, clinical examinations were performed only for students who had submitted the signed consent form.

### Clinical data collection

The dental status of all students was examined by a single dentist (BSP) in the school setting, using a WHO probe and a headlight. The data were recorded by a dental nurse on a structured data collection sheet. A portable air-powered device was also used to dry the teeth. Examinations were conducted based on the International Caries Detection and Assessment System (ICDAS) criteria [21]. This scoring system has been developed by an international group of researchers in 2007 to facilitate the assessment of enamel caries and active dentinal lesions. This system is based on a combination of factors including the clinical appearance of the lesion, its location in a plaque stagnation area, and the tactile sensation (texture) felt when a round-tipped probe is gently drawn across the tooth surface.

The ICDAS has proven to be reliable, offering greater sensitivity and accuracy in detecting carious lesions compared to other common indices such as DMFT. It is particularly suitable for epidemiological surveys involving children, as it allows for the monitoring of caries progression from early stages to advanced lesions [22]. In this system, two codes are assigned to each tooth surface; one indicating the current treatment status (e.g., filled with composite, amalgam, crown, extracted, or no treatment), and the other representing the stage of dental caries lesions, ranging from 0 to 6.

Therefore, the identification of dental caries on the coronal surface involved a two-step procedure. The first step was to categorize each tooth surface as sound, filled, restored, veneered, or missing. The second step was to classify the caries stage using an ordinal scale.

Accordingly, the number of non-cavitated carious lesions (d_1-2_: ICDAS codes 1–2), cavitated/dentine carious lesions (d_3-6_: ICDAS codes 3–6), filled cavities (f), and missing teeth due to caries (m) were recorded for each participant and six indices were calculated to evaluate the severity of caries in deciduous teeth: d_1-2_, d_3-6_, filled surfaces, missing surfaces, d_3-6_mfs, and d_1-6_mfs. Additionally, the following thresholds were considered: d_3-6_mfs ≥ 1 indicated children with dental caries; d_3-6_ ≥ 1, children in need of treatment; d_1-2_ ≥ 1, children with early caries; and d_1-6_mfs ≥ 1, children with any type of carious lesion [22].

The examiner’s performance in assessing the dental status of children according to ICDAS was calibrated through a standardized training process, which included completion of an online training course and successful participation in an online examination provided on www.iccms-web.com to ensure the intra examiner reliability.

### Determinants of children’s oral health

The study variables were selected based on the World Health Organization’s Social Determinants of Health (SDH) framework, which served as a conceptual roadmap [7]. According to this model, the elements pertaining to the “socioeconomic and political context” were treated as uniform, given that the study was conducted within a specific geographical region. Variables such as educational level, occupation, and income were evaluated as components of the family’s socioeconomic status.

Behavioral, biological, and psychosocial variables were also included to reflect intermediary determinants. The components were clearly operationalized by extracting indicators from a review of existing dental literature, followed by the development and validation of a structured questionnaire covering socioeconomic status, behavioral factors, and psychological factors (see S1 File).

### Child-related variables

The variables examined included the child’s gender, birth order, and oral hygiene practices as reported by parents. The report covered the number of dental visits in the past 12 months (0 = never, 1 = yes), the reason for the last dental visit (0 = treatment, 1 = check-up), the frequency of tooth cleaning (0 = never/a few times a month/once weekly, 1 = several times weekly, 2 = once or more daily), the tools used for cleaning teeth and gums (0 = none, 1 = floss, 2 = toothbrush, 3 = both), toothpaste use (0 = no, 1 = yes), and use of fluoride-containing toothpaste (0 = no/don’t know, 1 = yes).

These items were selected based on the World Health Organization’s Oral Health Survey: Basic Methods, 5th edition, which has been translated and validated in Persian and is available at https://iris.who.int/handle/10665/97035 [23]. The scores for each domain were aggregated to generate a composite score for oral hygiene practices. Consequently, each child was assigned a score ranging from 0 to 10. Scoring criteria were developed through consensus among four dental public health experts during a focused group session.

For example, a boy who had never visited a dentist (hence, no check-ups; score = 0), was reported to clean his teeth once a week (score = 0), used only a toothbrush (score = 2), used toothpaste (score = 1), but was unaware of its fluoride content (score = 0), would receive a total oral hygiene score of 3.

### Nutritional behaviors and habits

In this study, the frequency of sugary food consumption among children was recorded based on the WHO Basic Oral Health Survey Methods. Consumption frequencies were graded for various items as follows:

- Fresh fruit: 0 = rarely or never, 1 = several times monthly, 2 = once weekly, 3 = several times weekly, 4 = once daily, 5 = several times daily.
- Biscuits, cakes, cream cakes, baguette, cookies, etc.; jam and honey; sweets and candies: 10 = rarely or never, 8 = several times monthly, 6 = once weekly, 4 = several times weekly, 2 = once daily, 0 = several times daily.
- Lemonade, soda, or other sweet drinks; sugar-containing chewing gum: 5 = rarely or never, 4 = several times monthly, 3 = once weekly, 2 = several times weekly, 1 = once daily, 0 = several times daily.
- Milk with sugar; tea with sugar: 5 = rarely or never, 4 = several times monthly, 3 = once weekly, 2 = several times weekly, 1 = once daily, 0 = several times daily.

[23]. The total score for nutritional behaviors and habits was calculated by summing the individual item scores. Consequently, each child received a score ranging from 0 to 55.

For example, for a girl whose parents reported the following:

- Fresh fruits consumed daily (score = 4),
- Biscuits and cakes consumed several times monthly (score = 8),
- Lemonade, soda, or other sweet drinks consumed several times monthly (score = 4),
- Jam and honey consumed several times weekly (score = 4),
- Sugary gum, milk with sugar, and tea with sugar consumed rarely or never (score = 5 each),
- Sweets and candies consumed rarely or never (score = 10),

the total dietary behavior score would be 45.

### Family-related variables

The parents were asked about the type of school their child attended (public or private), the number of children in the family, the caregiver’s educational attainment (below diploma, diploma, or academic degree), and the caregiver’s employment status (housewife (mother), student/unemployed, retired, or employed in the public or private sector) [24]. The parents’ knowledge was assessed using an 18-item (true/false) questionnaire, developed based on our previous work [25]. This questionnaire was developed for use in primary schools. To ensure face and content validity, it was evaluated by five professors (two from the Department of Pediatric Dentistry and three from the Department of Public Oral Health). They were asked to rate the relevance of the questions and the proposed objectives based on a 3-point Likert scale (from 1: Completely relevant to 3: Not relevant). The reliability of the knowledge questions was assessed through a pilot study involving 50 parents attending the pediatric clinic, using the split-half method (r = 0.84).

The caregiver’s belief in oral health fatalism was assessed using a five-point Likert scale based on the statement: “Most children eventually develop dental cavities.” Responses were dichotomized as follows: 0 = neutral, disagree, or strongly disagree; 1 = agree or strongly agree [26]. They were also asked to complete the DASS-21 (Depression, Anxiety, and Stress Scale), which consists of 21 items, with seven items allocated to each subscale. Each item is rated on a four-point Likert scale (0 = not at all, 1 = to some degree, 2 = to a considerable degree, 3 = very much).

This study used the Persian version of the questionnaire, which demonstrated excellent internal consistency (Cronbach’s α > 0.90 for all three subscales), test-retest reliability above 0.80, and internal reliability above 0.78, as reported in a study by Asghari Moghaddam et al. (2008) [27]. They reported a strong correlation between DASS-21 scores and the Beck Depression Inventory (BDI) and the Four-System Anxiety Questionnaire (FSAQ). However, DASS-21 offers several advantages over these instruments, particularly in that its items do not overly emphasize somatic symptoms, which can often overlap with physical illness.

Additionally, the DASS-21 is a low-cost and user-friendly tool that enables the rapid assessment of depression, anxiety, and stress in both clinical and research settings [28]. The Persian version of the DASS-21 has been widely used across various Iranian populations and has demonstrated acceptable levels of validity and reliability [29–31].

To develop a comprehensive profile of participants’ psychological health based on the DASS-21 questionnaire and minimize potential multicollinearity among the three dimensions (Depression, Anxiety, and Stress), we combined the subscale scores through factor analysis to extract a single, unified factor score. This score was then incorporated into the regression models as an overall measure of psychological status.

### Statistical analysis

Quantitative variables were presented as means and standard deviations, while qualitative variables were expressed as frequencies and percentages. The normality of continuous data was assessed using the Kolmogorov-Smirnov test and Q-Q plots. Since several dental caries indices showed significant positive skewness, a logarithmic transformation was applied to normalize these variables and meet model assumptions. Initially, bivariate associations between each determinant (both child and parent variables as predictors) and dental indices (dependent variables) were evaluated. The relationship between quantitative determinants and dental indices was assessed using Pearson or Spearman correlation coefficients. The mean values of dental indices were compared across categories of categorical determinants using independent samples t-tests (or non-parametric Mann-Whitney U test) and analysis of variance (ANOVA) (or non-parametric Kruskal-Wallis test), with Bonferroni post hoc tests following ANOVA. The multivariable associations of determinants with quantitative dental indices (d_1-6_mfs, d_1-2_, d_3-6_, and d_3-6_mfs) were analyzed using linear regression. Additionally, the multivariable associations between factors and dichotomous dental indices, such as having missed teeth (yes/no), were analyzed using binary logistic regression. The number of filled teeth (categorized as 0, 1–10 = 1, 11–20 = 2, 21–30 = 3, > 30 = 4) as an ordinal dependent variable was analyzed using ordinal logistic regression. The results from linear regression were presented as regression coefficients (B) with standard errors, along with 95% confidence intervals (95%CI) for the regression coefficients. The results from logistic regression were expressed as odds ratios (OR) with 95% confidence intervals for OR.In our multiple regression models, all measured potential determinants were entered as independent variables, ensuring that the estimated coefficients for each determinant were adjusted for the presence of all others. Multicollinearity in multiple regression analysis was assessed using variance inflation factors (VIF). As suggested by O’Brien [32], VIF values above 5 indicate potential collinearity. We observed that all VIF values were below 5. All statistical analyses were performed using SPSS software version 20 (Released 2011. IBM SPSS Statistics for Windows, Version 20.0. Armonk, NY: IBM Corp).

## Results

A total of 417 signed consent forms were collected from the 551 questionnaires distributed to parents. Children were assessed across nine schools. The frequency distribution of parental demographic and psychological characteristics is presented in Table 1. Nearly equal proportions of girls and boys from both public and private schools participated in the study. The majority of parents had two children, possessed a university degree, and were employed (Table 1). The mean knowledge score of parents was 12.5 ± 3.2 out of 18, indicating that the majority exhibited good knowledge. The mean stress score was 8.9 ± 8.3. The mean scores for anxiety and depression were 7.92 ± 5.08 and 7.66 ± 5.43, respectively. They were mostly categorized as having a normal status. The mean total score for children’s health behavior was 6.36 ± 1.82 out of 10, while the mean nutritional behavior score (range 5–55) was 47.33 ± 8.22.

**Table 1:** Demographic, familiy and psychological characteristics of participated Parents (N = 417).

Variable	Frequency	percent
Child’s gender		
Boys	222.0	53.2
Girls	195.0	46.8
Type of school		
Public	226.0	54.2
Private	191.0	45.8
Number of children		
1	77.0	18.5
2	226.0	54.2
3	85.0	20.4
4	19.0	4.6
5	10.0	2.4
Child’s birth order		
1	180.0	43.2
2	172.0	41.2
3	53.0	12.7
4 or more	12.0	2.9
Head of household’s education level		
Under diploma	101.0	24.2
Diploma	97.0	23.3
Academic education	219.0	52.5
Head of household’s employment status		
Housewife (for women)/student/unemployed	81.0	19.4
Retired	8.0	1.9
Governmental/Self-employed	328.0	78.7
Fatalistic belief		
Neutral/disagree/strongly disagree	236.0	56.6
Agree/strongly agree	181.0	43.4
Knowledge Level		
Low	20	4.80
Moderate	164	39.30
Good	233	55.90
Stress Level		
Normal	336	80.60
Mild	35	8.40
Moderate	23	5.50
Severe	16	3.80
Very severe	7	1.70
Anxiety Level		
Normal	312	74.80
Mild	23	5.50
Moderate	43	10.30
Severe	17	4.10
Very severe	22	5.30
Depression Level		
Normal	335	80.30
Mild	23	5.50
Moderate	34	8.20
Severe	15	3.60
Very severe	10	2.40

Data are reported as frequency and percentage.

The relationship between quantitative determinants of children and parents and oral health indicators related to deciduous teeth is shown in Table 2. Results indicate that the d_1-6_mfs and d_3-6_mfs indices exhibited a significant inverse relationship only with the children’s nutrition score. The caries index (d_3-6_) had a positive relationship with the number of children, child’s birth order, and depression-anxiety-stress scores, while demonstrating a significant inverse relationship with oral and nutritional health behaviors and caregiver knowledge. The d_1-2_ and m indices showed a significant relationship with none of the variables. However, the f index demonstrated a significant inverse relationship with the number of children, child’s birth order, and depression-anxiety-stress scores, as well as a significant positive relationship with oral health behavior and caregiver knowledge (Table 2).

**Table 2:** Correlation coefficints of the association between family and child related factors and the oral health ststud measures.

	$d_{1-6}mfs$	$d_{3-6}mfs$	$d_{3-6}$	$d_{1-2}$	m	f
Number of children	−0.066	−0.023	0.159^**^	−0.098	0.057	−0.169^**^
Child’s birth order	−0.023	−0.016	0.193^**^	−0.051	0.053	−0.200^**^
Parents’ depression score	0.011	0.035	0.132^**^	−0.038	0.079	−0.166^**^
Parents’ anxiety score	0.054	0.084	0.181^**^	−0.037	0.095	−0.108^*^
Parents’ stress score	−0.034	0.032	0.143^**^	−0.089	0.082	−0.158^**^
Oral health behavior score of children	−0.003	−0.047	−0.186^**^	0.098	−0.004	0.152^**^
Sugar consumption by children	−0.106^*^	−0.145^**^	−0.125^*^	0.053	−0.068	−0.009
Parents’ knowledge score	−0.052	−0.035	−0.184^**^	0.038	−0.009	0.179^**^

Note-

**  = P-value<0.001,

*  = P-value<0.05

**Fig 1:**
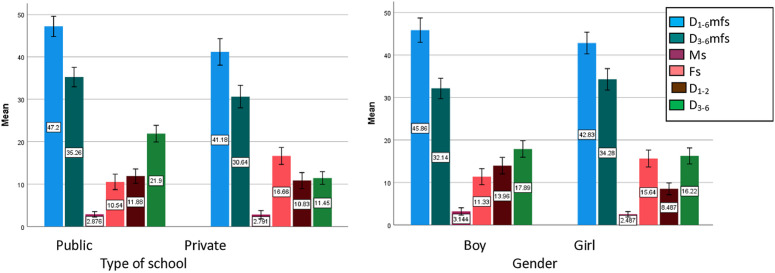
The distribution of indicators mean based on children gender and the type of their school (private/ public)- Error Bars: 95% CI.

Table 3 presents the mean scores of dental caries indicators for deciduous teeth categorized by qualitative determinants for both parents and children. Also, the distribution of indicators based on children’s gender and the type of their school (private/public) is presented in bar charts in Fig 1. Children enrolled in public schools had significantly higher scores in the d1-6mfs and d3-6mfs indices compared to those in private schools. The mean score of d1-6mfs was significantly higher in children who had a caregiver with a high school diploma or lower degree (post hoc, ANOVA) and those with an unemployed parent, a student, or a housewife (for women). The results also showed that the mean score of d_1-2_ was significantly higher in boys and children with a retired caregiver. The m index exhibited no significant associations with any variables; however, the f index was significantly higher among female children, those attending private schools, children with a university-educated caregiver, and those with a retired caregiver (Table 3).

**Table 3:** Comparison of oral health indices based on the gender, type of school, educational, occupation and fatalistic belief level of parents.

		$d_{1-6}mfs$	$d_{3-6}mfs$	$d_{3-6}$	$d_{1-2}$	Missing teeth	Filled teeth
Child’s gender	girl	42.83±18.12	34.28±17.89	16.22±13.33	8.49±9.97	2.49±4.49	15.64±14.15
	boy	45.86±21.72	32.14±18.16	17.89±14.77	13.96±14.87	3.14±6.58	11.33±14.24
	P value	0.126	0.228	0.228	<0.0001	0.241	0.002
Type of school	Public	47.20±18.20	35.26±17.41	21.90±15.05	11.88±12.96	2.88±4.84	10.54±13.94
	Private	41.18±21.84	30.64±18.49	11.45±10.44	10.83±13.25	2.79±6.59	16.66±14.14
	P value	0.002	0.009	<0.0001	0.412	0.879	<0.0001
Head of household’s education level	Under diploma	44.87±19.68	34.63±17.32	25.93±14.84	10.14±13.66	3.02±4.85	5.78±10.98
	Diploma	46.32±19.56	34.33±17.47	18.42±15.55	11.97±12.55	2.99±4.87	12.94±13.87
	Academic education	43.42±20.64	31.93±18.60	12.46±10.70	11.73±13.07	2.68±6.38	17.01±14.58
	P value	0.484	0.351	<0.0001	0.534	0.849	<0.0001
Head of household’s employment status	Housewife (for women)/student/unemployed	41.64±18.91	33.06±17.59	22.77±16.91	8.58±11.47	3.40±5.35	6.90±12.12
	Retired	54.87±13.21	35.38±21.68	19.63±13.38	19.50±12.76	.63±1.76	15.13±17.81
	Governmental/Self-employed	44.88±20.51	33.11±18.11	15.65±13.03	11.90±13.36	2.75±5.84	14.89±14.36
	P value	0.145	0.940	<0.0001	0.026	0.360	<0.0001
Fatalistic belief	Neutral/disagree/strongly disagree	43.32±20.76	31.96±17.95	16.19±13.83	11.31±13.29	2.56±4.78	13.29±14.48
	Agree/strongly agree	45.91±19.28	34.69±18.08	18.30±14.45	11.51±12.86	3.19±6.72	13.41±14.20
	P value	0.193	0.127	0.131	0.877	0.264	0.929
Whole population		44.44 ± 20.15	33.14 ± 18.04	17.11 ± 14.12	11.40 ± 13.09	2.84 ± 5.70	13.34 ± 14.34

Data are presented as mean±SD and P-values resulted from independent samples t-test (or non-parametric Mann-Whitney U test) or ANOVA (or non-parametric Krsuskal-Waallis).

The study results also indicated that d_1-6_mfs ≥ 1 was observed in 98.8% of participants (n = 412), d_3-6_ ≥ 1 was observed in 93.8% of participants (n = 391), d_3-6_mfs ≥ 1 was observed in 97.4% of participants (n = 406), and d_1-2_ ≥ 1 was observed in 85.6% of participants (n = 357). Moreover, the missing tooth surfaces (ms ≥ 1) and filled surfaces (fs ≥ 1) were found in 31.4% (n = 131) and 61.9% (n = 258) of participants, respectively.

Multiple linear regression analyses (Table 4) demonstrated that the mean score of d_1−6_mfs was significantly higher in boys (B = 6.99, P = 0.001) and children attending public schools (B = 11.35, P < 0.0001), while it was significantly lower in children with a housewife caregiver (B = −6.33, P = 0.021) and those who were following a healthier diet (B = −0.25, P = 0.029).

**Table 4:** Multivariable linear regression of association between determinants (family and child levels) and the quantitative dental indices including d1-6mfs, d1-2, d3-6, d3-6mfs.

	d_1-6_mfs	P-value	d_3-6_mfs	P-value	d_3-6_	P-value	d_1-2_	P-value
	B (95%CI for B)		B (95%CI for B)		B (95%CI for B)		B (95%CI for B)	
Gender of children		0.001		0.612		0.010		<0.0001
Boy	6.99(2.86_11.12)	0.001	−.98(−4.76_2.80)	0.611	3.49(0.82_6.16)	0.010	8.23(5.93_10.52)	<0.0001
Girl	Ref	.	.	.	.	.	.	.
Type of school of children		<0.001		0.061		<0.0001		<0.0001
Public	11.34(6.31_16.38)	<0.001	4.42(−0.19-_0.03)	0.061	7.51(4.56_10.47)	<0.0001	6.78(3.99_9.56)	<0.0001
private	Ref	.	.	.	.	.	.	.
Number of children in the family	−3.28(−6.54_-.02)	0.058	−1.07(−4.06_1.91)	0.483	−1.99(−4.02_.041)	0.055	−1.97(−4.26_.30)	0.089
Child’s birth order in the family	2.23(−1.31_5.78)	0.217	.30(−2.95_3.55)	0.856	2.67(0.57_4.76)	0.012	1.50(−0.87_3.88)	0.215
Head of household’s education level		0.188		0.888		0.057		0.027
Under diploma	−5.954(−12.42_.51)	0.071	−1.35(−7.29_4.57)	0.653	5.29(0.90_9.68)	0.018	−4.76(−8.32_-1.21)	0.009
Diploma	−1.635(−6.92_3.63)	0.544	−.95(−5.79_3.88)	0.700	1.56(−1.71_4.84)	0.349	−.78(−3.77_2.21)	0.608
Academic education	Ref	.	.	.	.	.	.	.
Head of household’s employment status		0.031		0.547		0.984		0.018
Housewife (for women)/student/unemployed	−6.33(−11.71_-0.95)	0.021	−2.73(−7.69_2.20)	0.277	−.22(−4.17_3.73)	0.913	−3.56(−6.77_-0.35)	0.029
retire	7.45(−6.19_21.11)	0.284	.37(−12.14_12.90)	0.953	.472(−6.51_7.46)	0.895	7.05(−2.09_16.19)	0.131
governmental/self-employed	Ref	.	.	.	.	.	.	.
Oral health behavior scores of children	−0.30(−1.40_0.79)	0.584	−.657(−1.66_0.34)	0.200	−.82(−1.57_-0.07)	0.031	0.34(−0.29_0.98)	0.295
Sugar consumption by children	−0.25(−0.48_-0.02)	.029	−.29(−0.50_-0.08)	0.006	−.10(−0.24_0.03)	0.142	0.03(−0.13_0.21)	0.719
Knowledge score		0.099		0.839		0.313		0.039
Low	6.98(−2.30_16.26)	0.141	−.29(−8.81_8.21)	0.945	3.11(−3.58_9.81)	0.362	6.56(−1.12_14.26)	0.094
Moderate	3.94(−.14_8.03)	0.059	1.05(−2.94_4.80)	0.581	1.80(−.81_4.41)	0.176	2.69(.22_5.17)	0.033
good	Ref	.	.	.	.	.		.
Fatalistic belief		0.333		0.288		0.721		0.776
neutral/disagree/strongly disagree	−1.87(−5.66_1.91)	0.333	−1.88(−5.35_1.59)	0.288	−.44(−2.88_1.99)	0.721	−.34(−2.73_2.03)	0.776
agree/strongly agree	Ref	.	.	.	.	.	.	.
Psychological status	−.42(−2.53_1.69)	0.695	.86(−1.07_2.80)	0.384	.50(−1.10_2.12)	0.53	−1.24(−2.54_0.05)	0.060

Data are regression coefficient-B (95%CI for B), Results and p-values obtained from multiple linear regression. Ref: = reference category

The study results revealed that the mean d_3-6_mfs was significantly lower in children following a healthier diet (B = −0.29, P = 0.006). In addition, the mean score of d_3-6_ exhibited a significant positive relationship with male gender (B = 3.49, P = 0.010), studying in public schools (B = 7.51, P < .001), child’s birth order (B = 2.67, P = 0.012), and elementary/junior high school educational attainment (B = 5.29, P = 0.018). By contrast, it demonstrated a significant negative relationship with oral health behavior (B = −0.82, P = 0.031). The d_1-2_ index demonstrated a significant positive relationship with male gender (B = 8.23, P < 0.001), studying in public schools (B = 6.78, P = 0.000), and moderate knowledge (B = 2.69, P = 0.033), and a significant negative relationship with elementary/junior high school educational attainment (B = −4.76, P = 0.009) and caregiver’s job (B = −3.56, P = 0.029) (Table 4). Logistic regression analysis showed that none of the determinants exhibited a significant relationship with the m index (missing teeth). The odds of having a filled tooth were significantly greater in girls (OR=2.337, P < 0.0001), children attending private schools (OR=1.909, P = 0.010), and children whose caregivers possessed a high school diploma (OR=1.901, P = 0.048) or a university degree (OR=2.470, P = 0.007) compared to those whose caregivers had elementary/junior high school educational attainment and children with an employed caregiver (OR=1.889, P = 0.021) (Table 5).

**Table 5:** Multivariable binary and ordinal logistic regression of the association between determinant (family and child levels) and the qualitative oral health indices (missing and filled teeth).

	Missing teeth OR (95%CI for OR)	P-value	Filled teeth OR (95%CI for OR)	P-value
Gender of children		0.805		<0.0001
Boy	1.06(.66_1.69)	0.805	Ref	.
Girl	Ref	.	2.33(1.51_3.59)	<0.0001
Type of school of children		0.725		0.010
Public	1.10 (.62_1.97)	0.725	Ref	.
private	Ref	.	1.90 (1.17_3.11)	0.010
Number of children	0.93 (0.65_1.34)	0.718	1.03 (0.75_1.40)	0.848
Child’s birth order	0.91(0.61_1.37)	0.669	0.73 (0.51_1.03)	0.103
Head of household’s education level		0.526		0.024
Under diploma	0.81 (0.38_1.70)	0.578	Ref	.
Diploma	0.71 (0.39_1.28)	0.257	1.90 (1.07_3.50)	0.048
Academic education	Ref	.	2.47 (1.28_4.74)	0.007
Head of household’s employment status		0.406		0.051
Housewife (for women)/student/unemployed	0.88 (0.47_1.63)	0.693	Ref	.
retired	3.98 (0.44_35.77)	0.217	3.37 (0.62_18.33)	0.159
governmental/self-employed	Ref	.	1.89 (1.10_3.23)	0.021
Oral health behavior scores of children	0.95 (0.84_1.07)	0.448	1.01 (0.90_1.13)	0.779
Sugar consumption by children	1.03 (0.97_1.03)	0.813	0.99(0.97_1.01)	0.471
Knowledge score		0.415		0.402
Low	1.04 (0.39_2.97)	0.935	Ref	.
Moderate	1.36 (0.85_2.18)	0.195	1.60 (0.46_5.40)	0.452
Good	Ref	.	2.01 (0.56_7.12)	0.278
Fatalistic belief		0.343		0.256
Neutral/disagree/strongly disagree	1.23 (0.80_1.88)	0.343	Ref	.
Agree/strongly agree	Ref	.	1.24 (0.85_1.81)	0.256
Psychological status	0.81 (0.63_1.04)	0.090	0.96 (0.79_1.18)	0.734

## Discussion

This study evaluated the relationship between factors influencing oral health, as per the WHO framework, and indices of deciduous tooth decay in Isfahan Province. We used a multistage sampling method to recruit a representative sample of schoolchildren. This method has also been used as an acceptable and reliable sampling method in previous studies conducted in Isfahan City among schoolchildren [25,33]. We did our best to include children with a good diversity according to their school setting (private and public) and a good coverage of the districts (3 out of 6) with an efficient sample size. The analyses indicated that deciduous tooth decay is associated with factors such as gender, type of school, birth order, occupation, diet, educational attainment, oral health behaviors, and knowledge. In our study, 93% of the children exhibited cavitated dental caries necessitating treatment.

The acquired statistics are consistent with those from the most recent national survey in Iran [34], indicating that only 7% of children in Isfahan Province and 8% nationwide exhibited no dental caries; however, these figures diverge from comparable data in numerous developed nations. The prevalence of dental caries in Brazil is reported at 66.3%, with 88% of affected teeth exhibiting active lesions [22]. A meta-analysis by Kazeminia *et al*. (2020) indicated that the prevalence of deciduous tooth decay was lower in Europe, Australia, and the Americas compared to Africa and Asia [35]. A study by Ana Armas-Vega et al. (2023) in Galápagos found that 1.9% (n = 15) of participants exhibited no carious lesions, while 98.1% (n = 789) presented with at least one type of carious lesion [36]. This result is consistent with the findings of this study.

This study demonstrated the association of demographic characteristics and socioeconomic factors. Boys exhibited a higher prevalence of dental caries and received the lowest level of restorative treatment. While some studies have reported a higher prevalence of dental caries in girls compared to boys [22], the results of others are consistent with the findings of this study [37]. This discrepancy may be attributed to biological factors, including genetics, hormones, and behavioral factors [38]. Another reason may be the greater propensity among girls to use dental services compared to boys, as well as cultural factors in Iran, where parents often prioritize the appearance of their daughters over that of their sons [39].

This study indicated that enrollment in public schools was associated with a higher incidence of dental caries in children and a lower rate of receiving restorative treatments, which is consistent with the findings of some previous studies [40–42]. The choice of parents to enroll their child in a private or public school is largely influenced by their socioeconomic status. Parents with higher socioeconomic status tend to prefer private schools due to better educational quality, greater resource availability, and a more supportive environment, which in turn enhance their child’s academic performance, health outcomes, and behavioral patterns [40]. Martins et al. (2014) concluded that interventions targeting lifestyle and behavioral factors, and those that increased annual budgets and improved access to dental services yield limited success in addressing health inequalities. They emphasized that changing individual behavior requires environmental modifications. Given that children spend a significant portion of their day at school, schools should actively participate in the development of public health policies and provide environments that promote healthy behaviors [42]. Unlike most public schools, private schools typically provide oral health education to both students and their parents [43]. A study by Nery et al. (2020) revealed a higher prevalence of high-risk behaviors among students in public schools compared to those in private schools. The findings highlight the need to address the social factors asoociated with the students’ behaviors and dental caries [44].

The study findings indicated that the caregiver’s educational attainment and employment status were among the factors associated with the prevalence of dental caries in children. Children whose caregivers had an educational attainment lower than a high school diploma exhibited fewer non-cavitated carious lesions. However, educational attainment below a high school diploma and unemployment among caregivers were significantly associated with a higher prevalence of cavitated carious lesions and a reduced likelihood of receiving restorative treatments. Furthermore, improved oral health behavior was associated with a lower prevalence of cavitated carious lesions. These findings are supported by previous studies [45], which indicate that oral hygiene and health behaviors, including regular tooth brushing and reduced sugar intake, are inversely correlated with the occurrence of superficial and non-cavitated carious lesions, independent of socioeconomic status. These findings suggest that caregivers with elementary or junior high school educational attainment are predominantly unemployed and spend most of their time at home, which may allow them to provide better care for their children, thereby reducing the incidence of tooth decay. However, there is a clear inequality in the incidence of cavitated carious lesions when considering factors such as caregivers’ educational attainment and other socioeconomic status indicators [45]. Caregivers with higher levels of education and income are more likely to obtain regular dental checkups and access dental services [43,46,47]. Conversely, low educational attainment can lead to lower income, unemployment, or unfavorable employment conditions, which may, in turn, influence health behaviors [48]. In contrast, more educated caregivers may use the internet and social media to access reliable information and understand the importance of teaching their children proper brushing techniques and maintaining good oral hygiene [43]. Given these findings, it appears that caregivers with elementary or junior high school educational attainment are more likely to be unemployed. Moreover, a consistent correlation has generally been observed between low health literacy and low socioeconomic status (particularly low educational attainment), which is a significant risk factor associated with health status, quality of life, health-related outcomes, health behaviors, and the use of preventive services [49]. It has been reported in previous studies that women with higher oral health literacy and knowledge levels also had a more favorable attitude toward their children’s oral health and demonstrated better personal oral health behaviors [25,50]. These findings highlight the importance of increasing mothers’ awareness and oral health education as an essential strategy in oral health programs, which has been emphasized repeatedly. Furthermore, it is vital to note that caregivers’—especially mothers’—misperceptions about their own oral health could result in fatalistic attitudes and hamper their children’s oral health behavior. In a study conducted in Iran, it was revealed that more than 70% of mothers of 9-year-old schoolchildren believed that it is natural for people to lose all their teeth in old age [51]. In another study in Iran, it was reported that the majority of mothers held misconceptions about the importance of care and treatment, especially for primary dentition. They believed that primary teeth are temporary and not worth treating [52]. These misconceptions might arise from cultural backgrounds, as studies from different nations with diverse cultures have reported varying beliefs about the value of primary dentition and identified it as a barrier to the utilization of preventive oral health care [53,54].

This study demonstrated that a healthy diet significantly reduced the mean scores of d₁–₆mfs and d₃–₆mfs. Many other studies have also reported similar findings, including the study conducted by de Melo et al. (2019), who identified the consumption of sweets between meals as a risk factor for an increased risk of dental caries in children with unhealthy eating habits [41]. A prospective cohort study by Peres et al. (2016) showed a higher prevalence of dental caries and a greater mean DMFT index in the high sugar consumption group compared to the low sugar consumption group, particularly among individuals aged 6–12 years [55]. Previous studies have confirmed the role of fermentable carbohydrates in the proliferation of cariogenic bacteria, leading to a drop in oral pH and subsequent enamel demineralization [37]. Notably, there is a significant and independent association between malnutrition resulting from inadequate economic status and tooth decay, which often leads to increased sugar intake as a compensatory mechanism for poor nutritional status. Consequently, children from low-income families face a higher risk of developing dental caries, along with other systemic conditions, in the future. Therefore, it is necessary to interrupt this harmful chain through the development of public interventions that limit the supply of junk food to children—especially around schools—and policies that promote children’s easy access to healthy foods [56].

This study found a significant and direct relationship between the child’s birth order and the incidence of dental caries, which is consistent with the findings of some previous studies [57,58]. In fact, older children may assume the responsibility of monitoring the health behaviors of their younger siblings for various reasons, resulting in diminished parental care for the latter [58]. However, some studies have reported no association between the child’s birth order and the incidence of dental caries [59–61].

Univariate analyses in this study revealed a correlation between poor caregiver psychological status and a higher risk of cavitated carious lesions, as well as a reduction in restored surfaces. A study by Davidsen et al. (2020) found that children of parents with severe mental illness were at a significantly higher risk of developing dental caries compared to children of other parents. These parents also demonstrated a lower level of preparedness for engaging in preventive child healthcare activities. It is hypothesized that individuals with mental health issues exhibit lower health literacy levels—due to socioeconomic disadvantages—as well as cognitive and functional impairments. This highlights the necessity for targeted support to mitigate health inequalities [62]. A study conducted in Australia by Seow et al., using the DASS, found that mothers of children with early childhood dental caries exhibited mild or higher levels of depression and anxiety compared to mothers in the control group [63]. Some other studies have reported no significant association between parental stress and early childhood dental caries [64], as well as no significant association between maternal anxiety levels and their children’s DMFT scores [61]. Other studies have reported contradictory results. Using the DASS, Gavic et al. (2018) demonstrated that the incidence of active caries in children was significantly and negatively correlated with parental depression and stress levels, and significantly and positively correlated with parental anxiety levels. Moreover, the mean DMFT score exhibited a significant negative correlation with parental depression [65]. Different findings in studies might be attributed to variations in samples, contexts, cultures, and methodologies. Moreover, the reason for the differing results may be due to the fact that performance (i.e., oral health behavior) might improve with a certain level of anxiety; however, if anxiety continues to increase, performance begins to decline [66]. Certain types of anxiety may lead mothers to provide better oral care for their children [67].

However, in our study, the inclusion of this variable in the multivariate regression models did not result in a significant change, indicating that the effects of other factors were more statistically significant. This finding has also been reported in previous studies [61] and it is claimed that, as caries is a multifactorial disease, many factors—such as the presence of cariogenic bacteria, diet, socioeconomic factors, and behavioral characteristics—might play a more important role in its development [67].

Univariate analyses revealed a significant inverse relationship between caregiver knowledge and the incidence of cavitated carious lesions, as well as a significant direct relationship with restored surfaces. Multivariate analysis also showed a relationship between moderate knowledge and early dental caries. A study by El Ashiry et al. (2021) indicated that increased maternal educational attainment correlates with greater knowledge of the risks associated with neglecting oral health and the related disorders that can adversely affect children. Consequently, more educated mothers are more likely to promote tooth brushing and the maintenance of oral hygiene among their children [43].

We used the WHO Social Determinants of Health (SDH) framework as a roadmap to identify the associated factors. A key element of the Commission on Social Determinants of Health (CSDH) framework is the emphasis placed on the socioeconomic and political contexts—the structural determinants of health inequalities. This includes all the social and political mechanisms that generate, reinforce, and maintain social hierarchies, such as macroeconomic policies, educational systems, labor markets, fiscal policies, and welfare and health systems [7].

An essential feature of the framework is the need to develop context-specific strategies that address both the structural and intermediary determinants of oral health inequalities. These strategies can operate at various levels, from the individual to the global. For oral health promoters working at the local level, strategies can be developed to focus on individuals and local communities [16]. Oral health-promoting schools are good examples of local efforts implemented in different parts of the world to increase awareness among teachers, students, and their parents about the value of oral health and proper oral hygiene practices. Their effectiveness has also been reported in increasing access to oral health promotion programs and dental care, especially among disadvantaged populations, and in reducing health inequalities [25]. Schools offer a valuable opportunity for oral health promotion, as children and their family networks are readily accessible. Moreover, implementing oral health interventions during this critical stage of physical and cognitive development can help establish lifelong sustainable behaviors, beliefs, and attitudes [68]. In addition, it has been proven that schools serve as effective settings for increasing parental knowledge and enhancing their ability to manage their children’s oral health behaviors at home. This approach can help address health inequalities associated with low educational attainment and health literacy among parents [25].

These schools have been successful in reducing the risk of developing new dental caries lesions by 30–50%. Studies have shown that children involved in comprehensive programs targeting children, parents, and teachers—such as those that include supervised tooth brushing at schools—demonstrated significantly better oral health outcomes. These include improved behavior, better gingival health, and enhanced oral health-related quality of life [69].

Dental public health policymakers and dental professional organizations can also influence change at higher levels, such as national and international policy development. Oral health policymakers must adopt an intersectoral approach, collaborating with a wide range of stakeholders. For instance, policy action aimed at creating more supportive social conditions and environments for oral health could include measures to improve the availability, accessibility, and affordability of oral health-promoting services and dental care. Examples of such policies include water fluoridation, food and nutrition policies that encourage healthier eating, and expanding insurance coverage. These efforts can help create a more supportive social environment to promote better oral health among disadvantaged populations [16].

Additional measures that can be implemented by governments include imposing taxes on sugar-sweetened beverages or foods with added sugars, or both, to reduce access to sugary foods and beverages. These measures would aim to improve the quality of food available in schools. Another approach is to create restrictions on advertising sugar-sweetened beverages and marketing them to children, a practice that has been banned in Iran since 1994 for TV ads. Launching public awareness campaigns, which evidence shows are effective when using multiple communication methods and consistent implementation, can also help reduce the consumption of unhealthy foods and beverages. Furthermore, using clear labels on food packaging that indicate sugar content, modifying food formulations, and reducing serving sizes, as well as offering alternatives to sugar-sweetened beverages, can all contribute to improving public health outcomes [70].

## Conclusion

The findings of this study indicate that the prevalence of tooth decay among children is concerning. Key factors associated with this issue included demographic characteristics, socioeconomic status (such as parental educational attainment and employment status), biological and behavioral traits of the child, as well as the knowledge and mental and emotional well-being of the caregiver. Given the significant impact of tooth decay in childhood, it is crucial to develop effective, evidence-based policies and strategies to improve children’s oral health.

### Limitations

Since this study employed a cross-sectional design, it faced the inherent limitations of such studies in interpreting causal relationships. The household income index was excluded from the analyses because nearly half of the participants did not respond to this item. However, previous studies in Iran have deemed this index unreliable for the Iranian population. To address this limitation, we used an asset-based index, and we plan to incorporate it in our upcoming publications that aim to assess the inequality in the prevalence of dental caries and its determinants among schoolchildren comprehensively.In addition, dietary and behavioral data were collected based on parental reports, which may introduce recall bias or may not accurately reflect the children’s actual behaviors. While other studies have reported a strong association between parental behavioral reports, such as nutritional status and tooth brushing habits, and the observed behaviors of children, it is recommended that future studies directly observe children’s oral health behaviors or ask them directly. However, it may be challenging for children to fully understand the questions and provide valid responses.

Another limitation of our study is that, although we included a broad spectrum of individual, familial, and social variables commonly used in this field, there may still be additional unmeasured confounders and cultural factors specific to the Iranian context that were not captured. These factors may influence the observed associations and potentially affect the generalizability of our results. Future studies should consider integrating a wider range of context-specific variables to better understand these influences. Additionally, the cross-sectional nature of our study prevents the establishment of causal relationships between the variables studied. While we included a comprehensive range of factors, it is possible that unmeasured confounders and cultural factors unique to the Iranian context may still influence the observed associations. Therefore, these findings should be interpreted with caution, and further longitudinal studies are needed to confirm the causality and generalizability of our results.

## Supporting information

S1 File Questionnaire_dental_caries. (DOCX)
