# Supplementary material for: Determinants of dental caries in deciduous teeth among Iranian school children: Evidence from a developing country
Source: PLoS One. 2025 Aug 8;20(8):e0327141. doi: 10.1371/journal.pone.0327141 (PMC12334034; doi:10.1371/journal.pone.0327141)
Supplement: S1 File — (DOCX) [file pone.0327141.s001.docx]

Dear parents;

The present questionnaire is to assess the factors in association with dental caries incidence among school children. Your answers will help us to plan oral health preventive strategies in the society. The participation in this survey is non-compulsory and the responses will remain confidentially with the principal investigators.

Thank you for your cooperation.
 Oral Public Health Department,

Dental school, Isfahan University of Medical Sciences, Phone:+983137925594

In addition, if you agree and sign this questionnaire, your child will have an oral examination by a dentist and her/his treatment needs will be defined.

   I…………., father/mother of …….. by signing this part will allow Dr Tahani and Dr Pezeshki to use the information of this filled out questionnaire, and will allow them to examine the oral health status of my child in the school.

Name and Family name……………….

Signature…………

**1.Name of the school: ……, 2.Type of school: private / public 3. Type of school: girls/boys**

**4.Name of the school: ….. 5.Number of your children :…., 6.Order of your child:…**

**7.Head of households’ education level:** Illiterate/ Elementary school/ Middle school/ Diploma Bachelor or Associate degree /Master of Sciences or PhD degree/ Religious education

**8.Head of households’ employment status:** Governmental/ Self-employed/ Housewife (for women)/ Retired/Unemployed/ Student

**9.House room number:** Without room/One/ two/ Three and more

**10.Car ownership**: yes/no

**11.Using notebook, laptop, or tablet in the house:**  yes/no

**12.Fun, pleasure, travel abroad (in the last two-years):** yes/no

13. **Head of households’ income:**

**Child-related variables**

**14. dental visits over the past 12 months**: never, yes

**15. reason for the last dental visit**: treatment, checkup

**16. frequency of tooth cleaning:** never/a few times a month/once weekly, several times weekly, once daily/twice or more

**17. tools used for cleaning teeth and gums**: none, floss, toothbrush, both

**18. toothpaste use**: no, yes

**19. fluoride-containing toothpaste**: no, yes

20. **How often do you eat or drink any of the following foods, even**

**in small quantities?**

|  | rarely or never, | several times monthly | several times weekly | once daily | several times daily |
| --- | --- | --- | --- | --- | --- |
| fresh fruit |  |  |  |  |  |
| biscuits, cakes, cream cakes, baguette, cookies |  |  |  |  |  |
| jam and honey; sweets and candies |  |  |  |  |  |
| lemonade, soda, or other sweet drinks |  |  |  |  |  |
| sugar-containing chewing gum |  |  |  |  |  |
| milk / tea with sugar |  |  |  |  |  |
|  |  |  |  |  |  |

| **21. Knowledge Questions** | yes | No | Don’t know |
| --- | --- | --- | --- |
| 1. Can bleeding from the gums while brushing be a sign of gum disease?  2. Can frequent consumption of soda dissolve tooth enamel?  3. Can early extraction of deciduous teeth cause irregular permanent teeth?  5. In children with infected deciduous teeth, is it more likely to have decayed permanent teeth?  6. Is hereditary the main cause of tooth decay?  7. Is tooth decay caused by microbial plaque?  8. Is gum disease caused by microbial plaque (accumulation of microbes on the surface of teeth)?  9. Doesn't eating sweet foods cause tooth decay?  10. Is brushing without toothpaste enough to prevent tooth decay?  11. Is fluoride good for dental health?  12. Is gargling with salt water or any other mouthwash enough to clean your teeth?  13. Does eating less sweets, chocolate, candy and other sugars help prevent tooth decay?  14. Does brushing your teeth regularly help prevent gum disease?  15. Can tooth decay or gum disease cause disease in other parts of the body?  16. What are the signs of dental caries? Brown or black discoloration/ pain during eating sugary or cold snacks/ tearing of the floss/ all could be  17. what is the best recommendation about the tooth brush bristles? It should be…. Soft/ hard/ no difference/ I don’t know |  |  |  |

**22. Psychological factors:**

In my opinion, "most children eventually develop dental cavities". strongly disagree, disagree, neutral, agree , strongly agree.

**23. DASS21**

Please read each statement and circle a number 0, 1, 2 or 3 which indicates how much the statement

applied to you over the past week. There are no right or wrong answers. Do not spend too much

time on any statement.

The rating scale is as follows:

0 Did not apply to me at all

1 Applied to me to some degree, or some of the time

2 Applied to me to a considerable degree or a good part of time

3 Applied to me very much or most of the time

1 (s) I found it hard to wind down 0 1 2 3

2 (a) I was aware of dryness of my mouth 0 1 2 3

3 (d) I couldn’t seem to experience any positive feeling at all 0 1 2 3

4 (a) I experienced breathing difficulty (e.g. excessively rapid breathing,

breathlessness in the absence of physical exertion) 0 1 2 3

5 (d) I found it difficult to work up the initiative to do things 0 1 2 3

6 (s) I tended to over-react to situations 0 1 2 3

7 (a) I experienced trembling (e.g. in the hands) 0 1 2 3

8 (s) I felt that I was using a lot of nervous energy 0 1 2 3

9 (a) I was worried about situations in which I might panic and make a fool

of myself 0 1 2 3

10 (d) I felt that I had nothing to look forward to 0 1 2 3

11 (s) I found myself getting agitated 0 1 2 3

12 (s) I found it difficult to relax 0 1 2 3

13 (d) I felt down-hearted and blue 0 1 2 3

14 (s) I was intolerant of anything that kept me from getting on with what I

was doing 0 1 2 3

15 (a) I felt I was close to panic 0 1 2 3

16 (d) I was unable to become enthusiastic about anything 0 1 2 3

17 (d) I felt I wasn’t worth much as a person 0 1 2 3

18 (s) I felt that I was rather touchy 0 1 2 3

19 (a) I was aware of the action of my heart in the absence of physical

exertion (e.g. sense of heart rate increase, heart missing a beat) 0 1 2 3

20 (a) I felt scared without any good reason 0 1 2 3

21 (d) I felt that life was meaningless 0 1 2 3
